# Challenge of coexisting type 2 diabetes mellitus and insulinoma: a case report

**DOI:** 10.1186/s13256-021-03047-2

**Published:** 2021-09-29

**Authors:** Joseph Singbo, Michael Locketz, Ian Louis Ross

**Affiliations:** 1 Department of Medicine J47 Old Main Building Division of Endocrinology and Diabetes, Groote Schuur Hospital, University of Cape Town, Observatory, Cape Town, 7945 South Africa; 2 Department of Histopathology, Groote Schuur Hospital, University of Cape Town, Anzio Road, Observatory, Cape Town, South Africa

**Keywords:** Recurrent hypoglycemia, Diabetes mellitus, Insulinoma, Endoscopic ultrasound

## Abstract

**Background:**

Insulinomas are rare clinical entities, but concurrent diabetes mellitus is even more uncommon, and the combination is easily missed. Recurrent hypoglycemia could be misconstrued as improved glycemic control. We present an unusual patient with type 2 diabetes and neuroglycopenia, with apparent improved glycemic control due to an insulinoma.

**Case presentation:**

A 54-year-old mixed ancestry man with a positive family history of type 2 diabetes mellitus was diagnosed with type 2 diabetes mellitus and hypertension 8 years prior to admission. He presented with episodes of abnormal behavior and hypoglycemia. Inappropriately high insulin and C-peptide concentrations were identified at the time of hypoglycemia. Despite poor adherence to his diabetic treatment, he had no target organ damage relating to diabetes, and his hemoglobin A1c (HbA1c) was 5.3%. A diagnosis of insulinoma was made, and he was started on diazoxide, with endoscopic ultrasound revealing a possible lesion in the pancreatic tail measuring 12 mm × 12 mm. A fine-needle aspiration biopsy could not be performed due to overlying splenic arteries and the risk of vascular perforation. An intraoperative ultrasound confirmed a 15 mm × 10 mm tumor in the pancreatic tail, necessitating a partial pancreatectomy and splenectomy, which were curative. A well-differentiated intermediate grade 2 pancreatic neuroendocrine tumor producing insulin was confirmed on histopathology.

**Conclusions:**

Recurrent, progressive hypoglycemia and improved glycemic control in a diabetic, without an alternative explanation, may suggest an insulinoma. Insulinomas that exist with type 1 diabetes mellitus, particularly malignant insulinomas, must have escaped autoimmune attack through lack of autoantigen expression. Computed tomography on its own may be insufficiently sensitive for diagnosis of insulinomas, whereas endoscopic and intraoperative ultrasonography may improve the identification of the culprit lesion.

## Background

Hypoglycemia accounts for significantly increased mortality rates in diabetes. The coexistence of insulinoma and diabetes is so rare that there are only a few published case reports. In addition, there has been a case of insulinoma in which, despite stopping sulfonylureas, hypoglycemic episodes persisted [1]. We report a rare case of insulinoma in a 54-year-old man with pre-existing type 2 diabetes mellitus and a significant family history of diabetes. Despite poor adherence to therapy, he had unexplained well-controlled diabetes, hemoglobin A1c ( HbA1c) of 5.3 %, and recurrent hypoglycemic episodes, predominantly in the fasting state. As the diagnosis remains challenging, especially among diabetics, insulinoma is frequently diagnosed postmortem, due to the apparent optimal glucose control [2]. When hypoglycemic unawareness is present, it may be life-threatening, since it can induce irreversible brain damage, subsequent cardiac arrhythmias, and death.

Hypoglycemia is common, with potentially fatal consequences. The estimated rate of severe hypoglycemia events (needing emergency services) per patient per year ranges from 0.90 to 1.50 in patients with type 1 diabetes, but much lower in type 2 diabetes (from 0.30 to 0.63 per year) [3], where it is associated with an advanced clinical course and endogenous insulin deficiency. Hypoglycemia is associated with poorer quality of life and has significant financial implications. Complications of diabetes, malabsorption, associated endocrinopathies (for example, primary hypoadrenalism), factitious use of insulin, and psychological factors can predispose to hypoglycemia. The treating physician should always consider overly tight glycemic control, renal insufficiency, and pregnancy, and that previous hypoglycemia begets a future of hypoglycemia.

## Case presentation

A 54-year-old married mixed ancestry man and father of four children, working in the construction industry, was diagnosed simultaneously with type 2 diabetes mellitus and hypertension 8 years prior to admission. He also had a significant family history of type 2 diabetes mellitus, with his father and two of his brothers affected, all of whom are deceased, having suffered complications of ischemic cardiac failure and renal failure, respectively. However, none of the siblings had a history of admission for recurrent hypoglycemia. The father of the index subject died at 70 years of age, having had ischemic cardiomyopathy, which necessitated coronary artery bypass graft surgery prior to this event, whereas his two brothers died from diabetic nephropathy at age 48 and 50 years. In addition, there was no family history suggestive of pituitary disease or hyperparathyroidism. Between 2010 and 2017, our patient presented several times to his primary care physician with blood glucose measurements greater than 26 mmol/L. At each of these visits, he received short-acting human insulin and intravenous fluid. His final presentation to the primary health care facility with hyperglycemia occurred 12 months prior to admission.

His current presentation on June 27, 2018, involved a referral to his secondary care hospital, having been asymptomatic for a year, with no episodes of hyperglycemia except for weight gain of 14 kg in the preceding 3 months. According to his family, he suffered from confusion and sleepwalking, particularly in the early hours of the morning between 03:00 and 08:00. These symptoms occurred almost every day, associated with generalized body weakness and sweating, which were noticeable after each episode. His family reported physical and verbal aggression and confusion. During additional episodes in hospital, he was noted to be combative with the nursing staff and fellow patients and was found to be hypoglycemic on several occasions. Our patient appeared to develop symptoms indicative of hypoglycemic unawareness, as he manifested with no sympathetic symptoms, despite glucose concentrations below 1.8 mmol/L (3.8–5.5 mmol/L). During this admission, he exhibited recurrent episodes of fasting and post-prandial hypoglycemia, which measured between 1.2 and 3.0 mmol/L.

The possibility of nonconvulsive seizures was entertained; thus a computed tomography (CT) brain scan was ordered and found to be normal, whereas an electroencephalogram was not available at the secondary care facility. He was transferred to a tertiary hospital for evaluation by the endocrine service.

At admission, his chronic medications were metformin, enalapril, hydrochlorothiazide, and simvastatin**.** Aside from metformin, he was not taking any oral hypoglycemic agent or insulin and denied using any other agents. Pharmacy records failed to identify that he had received either a sulfonylurea or insulin. The two-hourly ward glucose measurements showed recurrent hypoglycemic episodes ranging from 1.2 to 3.4 mmol/L, occurring between 03:00 and 08:00.

Clinical examination failed to identify insulin injection sites or evidence of target organ damage relating to diabetes, hypertension, or any other chronic disease. In particular, he had no indication of melanoderma.

## Investigations

In view of the fasting hypoglycemia, the results of the investigations are shown in Table 1. In summary, the biochemical tests showed episodes of hypoglycemia with inappropriately high insulin and C-peptide levels, suggesting that the cause may be endogenous insulin secretion. The sulfonylurea screening was negative.

**Table 1:** Results of appropriate investigations directed at determining the cause of hypoglycemia

	Plasma glucose (4–7 mmol/L)	C-peptide (1.1–2.4 ug/L)	Insulin levels (2.6–24.9 mIU/L)	Sulfonylurea screening	Ketones
27/06/18	–	8.6	–	Rejected	–
28/06/18	2.6	5.5	40.7	Rejected	–
30/06/18	1.3	–	20.3	–	–
07/04/18	–	3.6	14.3	–	–
07/09/18	2.3	–	–	–	Negative
07/10/18	2.4	3.6	14.8	–	–

His HbA1c was 5.3%; the renal and hepatic function, serum calcium and phosphate levels, serum cortisol level (8:00 am), parathyroid hormone (PTH), pituitary function tests, and metanephrines were normal. His cortisol rose from 109 to 556 nmol/l within 30 minutes of a 250-microgram Synacthen^®^ test, excluding primary hypoadrenalism. The serum potassium and sodium were 3.2 and 138 mmol/L, respectively Insulin-like growth factor 1 (IGF-1) was 215.1 mU/L (55–248 mU/L). Liquid chromatography–tandem mass spectrometry of plasma and urine were negative for the following sulfonylureas: glimepiride, gliclazide, glyburide, glipizide, and gliquidone.

**Fig. 1:**
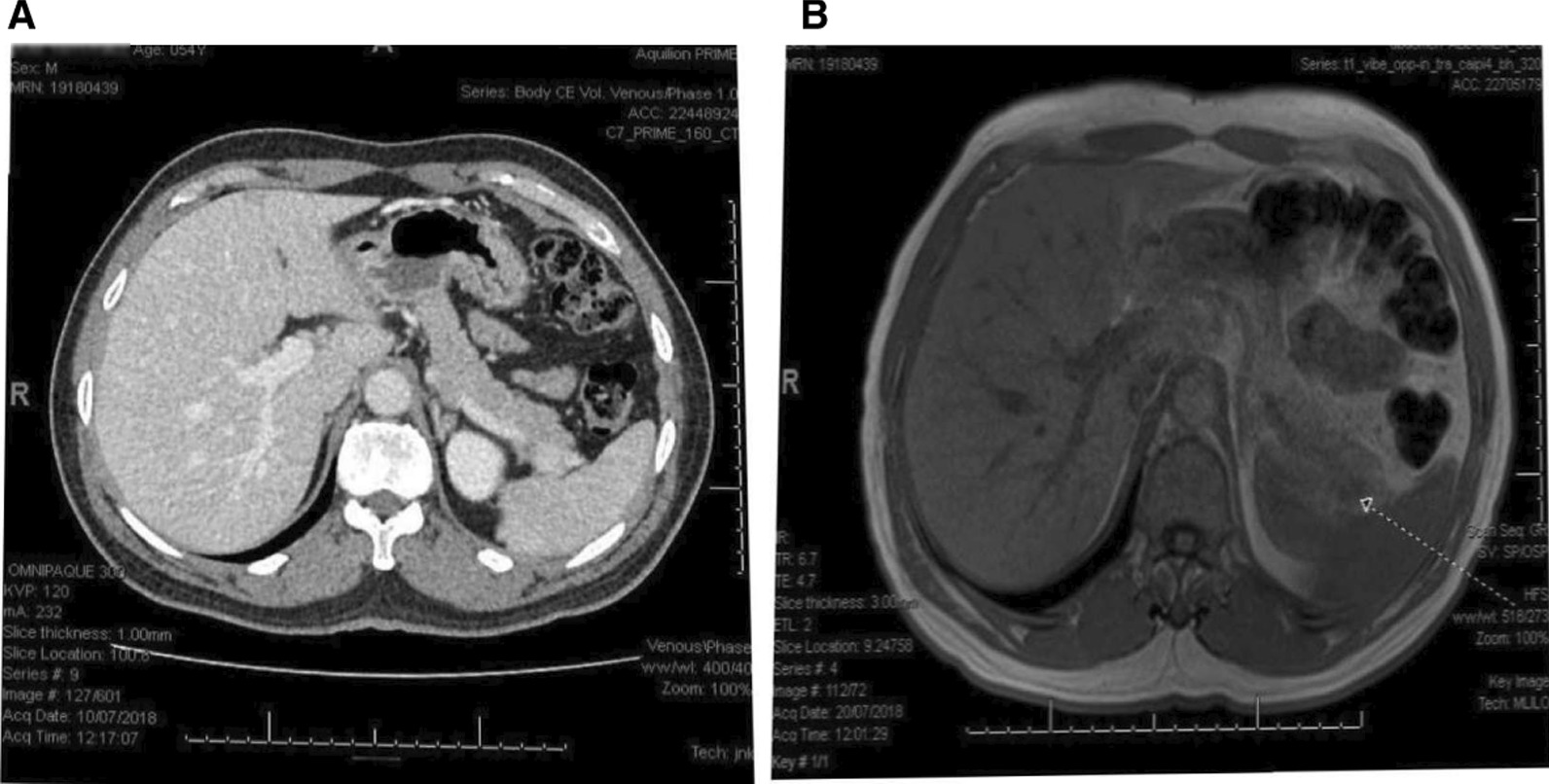
Contrast-enhanced computed tomography (CT) of the abdomen and magnetic resonance cholangiopancreatography (MRCP) of our patient. **A** CT of the abdomen showing a 12 × 10 mm mass lesion of the tail of the pancreas. **B** MRCP showing 10 × 12 mm mass in the pancreatic tail in keeping with an accessory spleen (arrow is indicating the lesion)

Given the prior history of type 2 diabetes, recent onset of symptomatic fasting hypoglycemia, and the inappropriately elevated serum insulin and C-peptide concentrations, a diagnosis of insulinoma was considered. A CT scan of the abdomen revealed a lesion measuring 12 mm × 10 mm in the tail of the pancreas (see Fig. 1A). Magnetic resonance cholangiopancreatography (MRCP) revealed a lesion measuring 10 mm × 12 mm which suggested an accessory spleen in the pancreatic tail (see Fig. 1B).

**Fig. 2:**
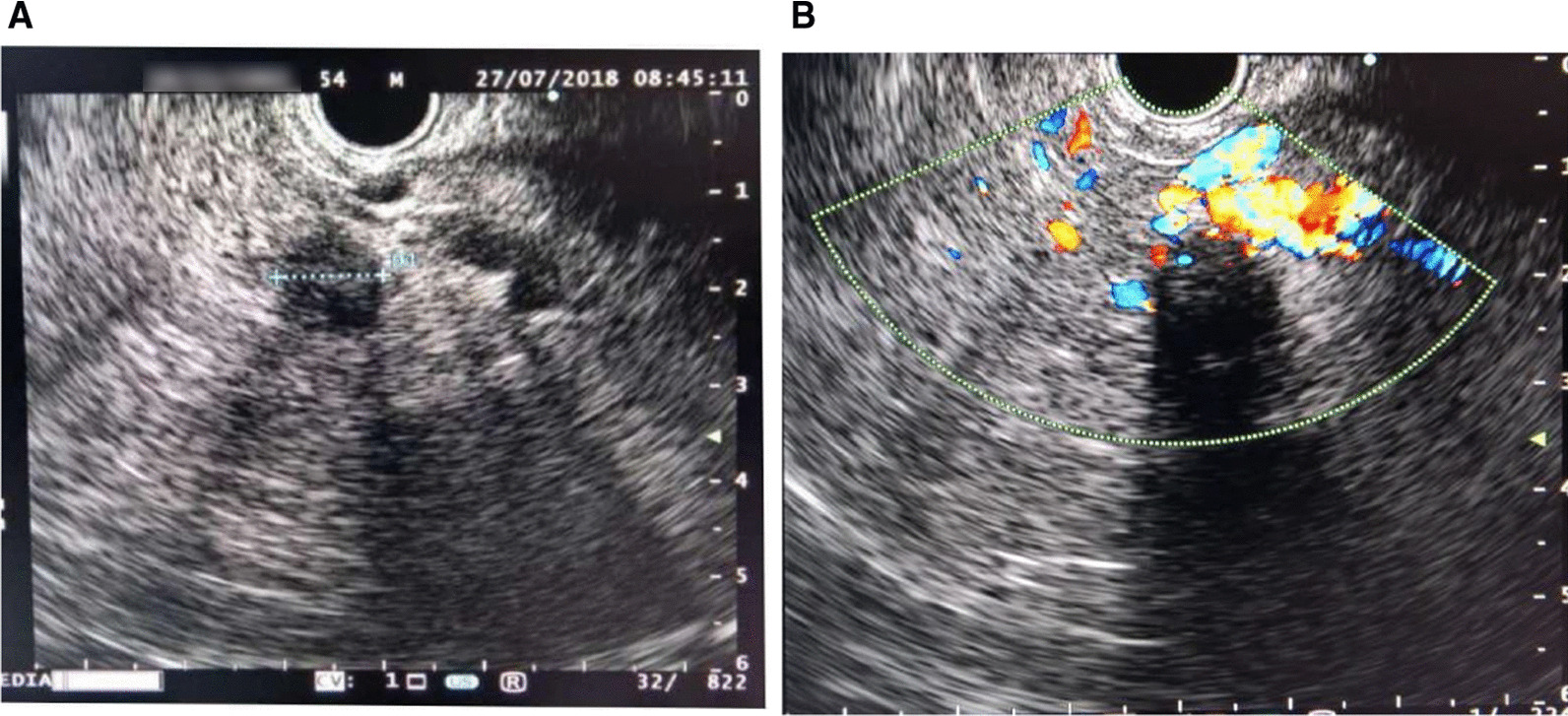
**A** Endoscopic ultrasound of our patient showing a 12 × 12 mm mass lesion of the pancreatic tail; **B** endoscopic ultrasound showing a mass lesion of the pancreatic tail with overlying splenic arteries (risk of perforation on fine-needle biopsy)

In view of the discordant reports on abdominal CT and MRCP, an endoscopic ultrasound was performed, which suggested a 12 mm × 12 mm lesion in the pancreatic tail (see Fig. 2A). A fine-needle aspiration biopsy was not performed due to overlying splenic arteries and the risk of vascular perforation (see Fig. 2B).

**Fig. 3:**
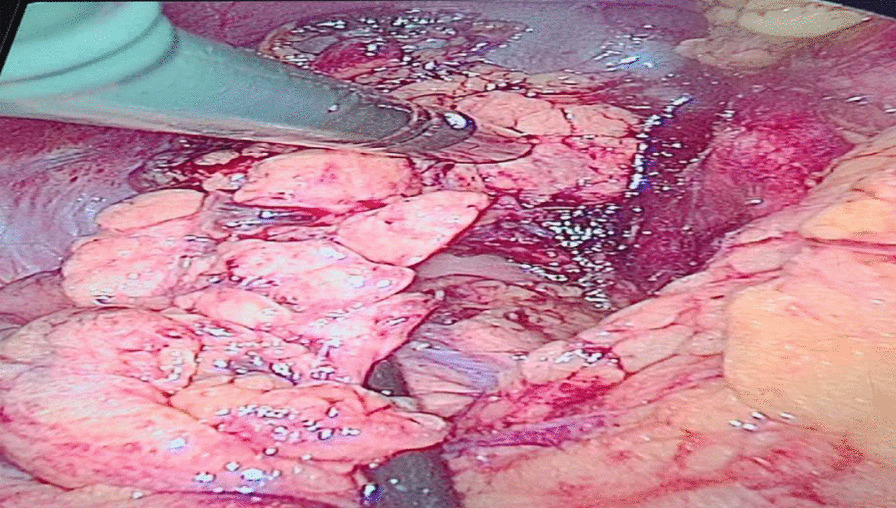
Intraoperative ultrasonography, searching for the tumor in our patient. (Dr. Sean Burmiester). Histopathological diagnosis.

**Fig. 4:**
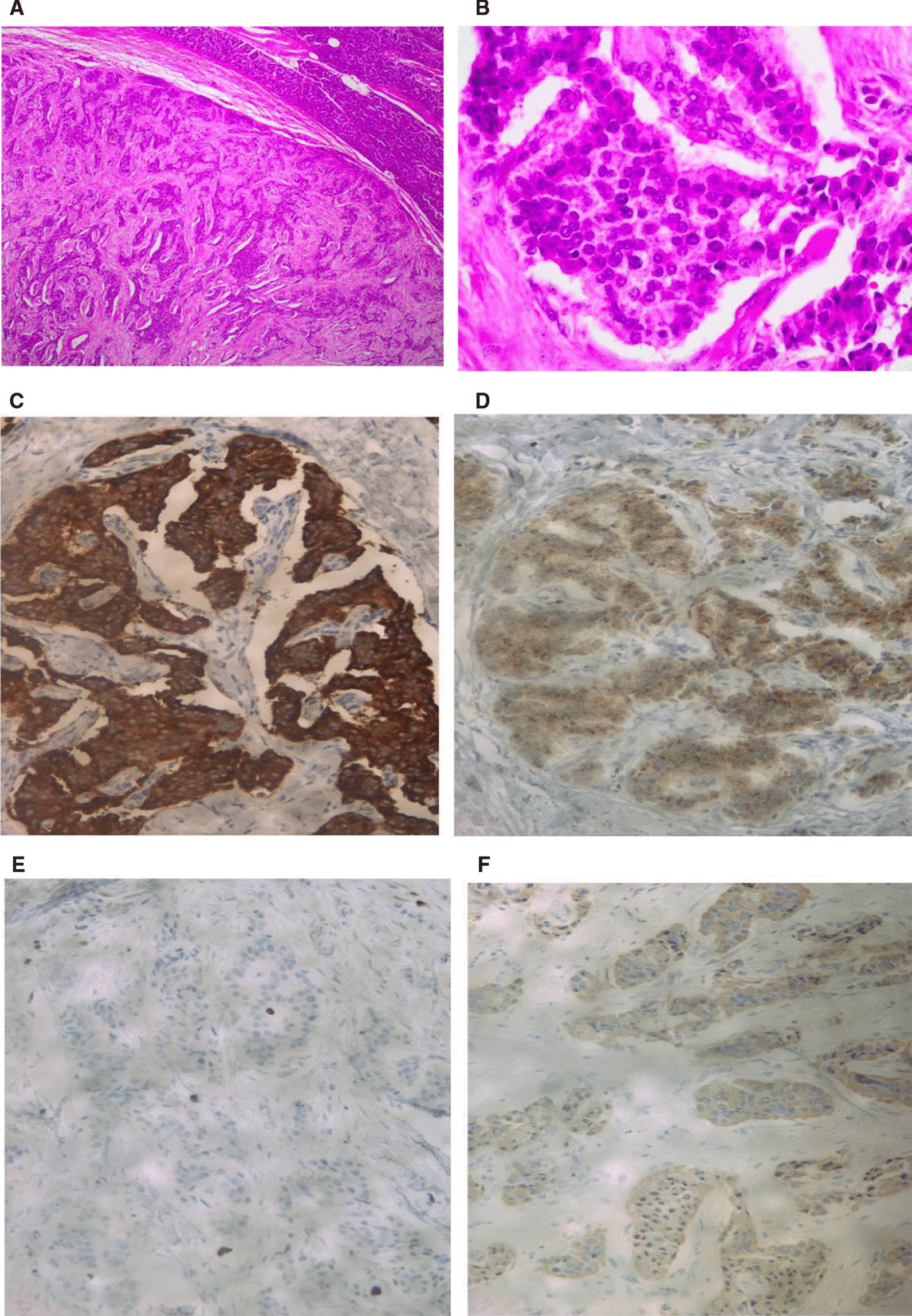
Histology of tail of pancreas excision showing a well-differentiated neuroendocrine tumor (NET) grade 2. **A** Hematoxylin and eosin stain, low-power magnification; **B** hematoxylin and eosin stain, high-power magnification; **C** immunohistochemical stain for synaptophysin, medium-power magnification; **D** immunohistochemical stain for chromogranin, medium-power magnification; **E** immunohistochemical stain for Ki67, medium-power magnification; **F** immunohistochemical stain for insulin, medium-power magnification

In view of the increasing number of hypoglycemic episodes and unawareness, diazoxide 75 mg was initiated and titrated up to 100 mg every 8 hours. An intraoperative ultrasound confirmed a 15 mm × 10 mm tumor in the pancreatic tail, necessitating a partial pancreatectomy and splenectomy (see Fig. 3). The histopathologic findings were in keeping with a well-differentiated pancreatic neuroendocrine tumour, grade 2, producing insulin (see Fig. 4). The well-circumscribed, 12-mm-diameter tumor was composed of nests and ribbons of cuboidal epithelial cells with small round central nuclei, speckled chromatin, and moderate amounts of granular eosinophilic cytoplasm, in a fibrous stromal background. There was no necrosis. The tumor was confined to the pancreas, with no lymphovascular or perineural invasion seen. There were zero mitotic figures per 10 high power fields, but the Ki67 proliferation index was 3%, corresponding to grade 2 of the World Health Organization (WHO) 2017 classification. Immunohistochemistry showed strong positive staining for synaptophysin, and weak positive staining for chromogranin A and insulin.

Postoperatively, our patient exhibited no further hypoglycemic episodes, and fasting and random blood glucose concentrations on metformin varied between 6.0 and 8.0 mmol/L. A pneumococcal vaccine was administered as prophylaxis for overwhelming postoperative streptococcal infection (OPSI).

## Discussion

We present our patient with type 2 diabetes mellitus, significant family history of type 2 diabetes, and recent onset of recurrent symptomatic hypoglycemia due to an insulinoma. The insulinoma most certainly modified his diabetic control, such that he did not need either oral hypoglycemic agents or insulin.

The association between diabetes and insulinoma is rare. In a retrospective study from the Mayo Clinic comprising 313 cases of confirmed insulinoma between 1927 and 1992, there was only one patient with pre-existing diabetes [4]. A Japanese review of 443 cases of insulinoma between 1976 and 1990 identified only one patient who had concurrent diabetes and insulinoma [5]. In a review from Taiwan of 23 cases of insulinoma spanning 22 years, only one patient had type 2 diabetes [5]. There are a few case reports of insulinoma in patients with type 1 diabetes. Insulinoma may mask the existence of type 1 diabetes or cause recurrent hypoglycemia and decreased insulin requirement in a type 1 diabetic patient [6, 7].

In 1962, a report suggested an association between insulinoma and a family history of diabetes [7], and about 30% of patients with insulinoma had a family history of diabetes in a series of the Mayo Clinic [8]. A possible explanation for a positive family history of diabetes may be insulin resistance and, consequently, exuberant elaboration of insulin. The increase in insulin secretion can theoretically result in hyperplasia of islet of Langerhans cells, which maintains a relative normoglycemic state. However, it is conceivable that without appropriate control mechanisms and a degree of autonomy, an insulin-secreting beta cell tumor could develop [9].

An insulinoma may also mask diabetes. In some case reports of insulinoma with concurrent diabetes mellitus, the diabetes was only diagnosed after the tumor was resected [5, 9]. There are also case reports where insulinomas were only diagnosed on postmortem examination of diabetes patients [3]. Overall, the coexistence of diabetes mellitus and insulinoma is rare, and there are no reported cases in South Africa. We recommend examining patients for primary or secondary hypoadrenalism, IGF-1, C-peptide, ketones, sulfonylurea concentrations, and in some instances insulin in cases of unprovoked hypoglycemia in a diabetic.

The occurrence of persistent hyperglycemia following surgical removal of the insulinoma suggests underlying diabetes, bearing in mind the possibility of damage to the pancreas during the surgical procedure and giving rise to diabetes, rather than a prior diagnosis of diabetes. In cases of coexistent type 1 diabetes mellitus, the blood tests reveal low levels of serum C-peptide and high titers of anti-glutamic acid decarboxylase antibody; histological examination of the resected specimen may reveal insulitis in non-tumorous pancreatic tissue in which beta cells had already disappeared [6]. In addition, there was infiltration of the insulinoma by inflammatory cells, as if it were insulitis of type 1 diabetes, suggesting the existence of anti-islet autoimmunity [6]. The absence of both proven autoimmunity and inflammatory infiltration together with adequate glycemic control on oral hypoglycemic agents suggests type 2 diabetes mellitus. A prolonged supervised fast is a gold standard test to evoke hypoglycemic episodes and is also useful in patients with diabetes mellitus [10]. This was not performed in our patient, as he manifested with unprovoked hypoglycemia. Considering their relative accuracy, continuous glucose monitoring devices may replace the prolonged fast in the near future. As expected in benign insulinoma, we did not encounter local tumor effects (local, regional organ or vascular compression), as these are described very rarely.

The majority of insulinomas are benign, whereas between 5 and 12% are malignant [9]. Only 22 cases have been published demonstrating the association between malignant insulinoma and diabetes [11]. There is no difference in the pattern of recurrence of hypoglycemia at presentation of patients with benign versus malignant insulinoma. However, we expect hypoglycemia to be more frequent and severe in the presence of malignant insulinoma depending on the size of the tumor and presence of metastases. A case report demonstrated paradoxical weight gain in a subject who had both type 2 diabetes mellitus and malignant insulinoma, and the explanation for the weight gain lies in recurrent eating to avoid hypoglycemia [10]. Distinction between malignant and benign tumors can only be made by the presence of metastasis, as there are no specific morphologic, biochemical, or genetic features distinguishing them. Histologically, insulinomas are epithelial neoplasms associated with strong and diffuse immunohistochemical expression of neuroendocrine markers such as synaptophysin and chromogranin. Most patients with malignant insulinoma have lymph node or liver metastases and, rarely, bone involvement [10].

It has been proposed that the insulin-producing cells in a malignant insulinoma and type 1 diabetes suggest that the insulin-producing malignant cells must have escaped autoimmune attack that otherwise had completely destroyed the beta cells of the patient [6]. No evidence of an inflammatory response in the resected tumor or normal pancreatic tissue could be identified [6]. It is speculated that a lack of auto-antigen expression in the insulinoma cells or their ability to escape immune surveillance in other ways accounts for an absence of inflammatory response, particularly in coexisting type 1 diabetes mellitus.

In our case and most of the available case reports of associated insulinoma and diabetes, hypoglycemia was successfully treated on diazoxide, and the surgical resection of the tumor was curative [9]. In instances where malignant insulinomas did not respond to surgical resection, alternative therapies such as peptide receptor radionuclide therapy (PRRT), after failure of everolimus and chemotherapy (streptozocin and capecitabine), have been used to achieve remission. On the other hand, there are reported cases of malignant insulinoma refractory to diazoxide and long-acting somatostatin analog using lanreotide [12]. By contrast, pasireotide, a multi-receptor-targeted somatostatin analog, exhibits a strong inhibitory effect of insulin secretion, and this has been documented to be effective in medical management of insulinoma [12].

Malignant insulinomas confer a truncated life expectancy after diagnosis, and substantially shorter if there are metastases. The major sites of metastasis or recurrence are the liver and regional lymph nodes [10]. The coexistence of diabetes mellitus and malignant insulinoma may result in a delay in diagnosis, but there is no evidence to suggest that it worsens the prognosis.

## Conclusions

We report an unusual association of type 2 diabetes mellitus and insulinoma. The apparent improvement in glycemic control and development of hypoglycemia, despite not using any hypoglycemic therapy, indicated a sinister underlying cause. Insulinomas are rare, especially among patients with type 2 diabetes mellitus, and there is a heavy reliance on CT scans of the abdomen to aid in diagnosis. It is acknowledged that intraoperative ultrasound may not always be available and may be impractical for smaller lesions. In cases when these lesions are suspected, they should be referred to a tertiary center for evaluation. Successful resection of the benign insulinoma eliminated further hypoglycemic episodes and was curative, whereas malignant insulinomas confer a poor prognosis.

## Data Availability

The authors grant permission for the use and dissemination of all enclosed data and material. The case report contains data pertaining to the patient’s management, but we do not have any data sets other than the repository housed by the Chemical Pathology Laboratory (National Health Laboratory Services Observatory Cape Town), which performed routine analyses. Hard copies and transcripts of the laboratory results can be forwarded to the Journal if necessary.

## References

[1] Ouleghzal K, Ziadi T, Menfaa M, Safia S. Association of insulinoma and type 2 diabetes mellitus. Int J Endocrinol Metab. 2016;15(1):e39439. 10.5812/ijem.39439 PMC5554613 28835758

[2] Kunieda T, Yamakita N, Yasuda K. Insulinoma in a patient with type 2 diabetes mellitus proved at autopsy. Endocr Pract. 2012;18:1038. 10.4158/EP12082.LT 23246687

[3] Nunez M, Dıaz S, Dilla T, Reviriego J, Perez A. Epidemiology, quality of life, and costs associated with hypoglycemia in patients with diabetes in Spain: a systematic literature review. Diabetes Ther. 2019;10:375–92. 10.1007/s13300-019-0563-0. PMC6437233 30661221

[4] Kane LA, Grant CS, Nippoldt TB, Service FJ. Insulinoma in a patient with NIDDM. Diabetes Care. 1993;16:1298–300. 10.2337/diacare.16.9.1298 8404437

[5] Ishii H, Ito T, Moriya S, Horie Y, Tsuchiya M. Insulinoma–a statistical review of 443 cases in Japan. Nihon Rinsho. 1993;51(Suppl):199–206. 8459545

[6] Lei WY, Wang TE, Chen TL, Chang WH, Yang TL, Wang CY. Insulinoma causing hypoglycemia in a patient with type 2 diabetes. J Formos Med Assoc. 2007;106:392–6. 10.1016/S0929-6646(09)60324-7 17561474

[7] Oikawa Y, Katsuki T, Kawasaki M, Hashiguchi A, Mukai K, Handa K, Tomita M, Kabeya Y, Asai Y, Iwase K, *et al*. Insulinoma may mask the existence of type 1 diabetes. Diabet Med. 2012;29:e138-141. 10.1111/j.1464-5491.2012.03615.x 22356209

[8] Gjelberg HK, Hoem D, Verbeke CS, Eide J, Cooper JG, Molven A. Hypoglycemia and decreased insulin requirement caused by malignant insulinoma in a type 1 diabetic patient: when the hoof beats are from a zebra, not a horse. Clin Case Rep. 2017;5:761–8. 10.1002/ccr3.927 PMC5457997 28588806

[9] Priestley JT. Hyperinsulinism. Ann R Coll Surg Engl. 1962;31:211–28. PMC2413456 13986185

[10] Ademoglu E, Unluturk U, Agbaht K, Karabork A, Corapcioglu D. Type 2 diabetes mellitus in a patient with malignant insulinoma manifesting following surgery. Diabet Med. 2012;29:E133–7. 10.1111/j.1464-5491.2012.03603.x 22313044

[11] Okabayashi T, Shima Y, Sumiyoshi T, Kozuki A, Ito S, Ogawa Y, Kobayashi M, Hanazaki K. Diagnosis and management of insulinoma. World J Gastroenterol. 2013;19:829–37. 10.3748/wjg.v19.i6.829 PMC3574879 23430217

[12] Hirshberg B, Cochran C, Skarulis MC, Libutti SK, Alexander HR, Wood BJ, Chang R, Kleiner DE, Gorden P. Malignant insulinoma: spectrum of unusual clinical features. Cancer. 2005;104:264–72. 10.1002/cncr.21179 PMC4136659 15937909

[13] Jawiarczyk A, Bolanowski M, Syrycka J, Bednarek-Tupikowska G, Kaluzny M, Kolodziejski A, Domoslawski P. Effective therapy of insulinoma by using long-acting somatostatin analogue. A case report and literature review. Exp Clin Endocrinol Diabetes. 2012;120:68–72. 10.1055/s-0031-1287792 22187292

